# Using the 12-item short-form health survey (SF-12) to evaluate self-rated health in an environmental justice community

**DOI:** 10.1186/s13690-024-01417-y

**Published:** 2024-10-18

**Authors:** Leanne S. Fawkes, Taehyun Roh, Thomas J. McDonald, Jennifer A. Horney, Weihsueh A. Chiu, Garett T. Sansom

**Affiliations:** 1 Department of Environmental and Occupational Health, Texas A&M School of Public Health, 212 Adriance Lab Rd., College Station, TX 77843 USA; 2 Department of Environmental and Occupational Health, University of Texas School of Public Health San Antonio at the University of Texas Health Science Center at San Antonio, San Antonio, TX 78229 USA; 3 Department of Epidemiology & Biostatistics, Texas A&M School of Public Health, 212 Adriance Lab Rd., College Station, TX 77843 USA; 4 Epidemiology Program, University of Delaware, College of Health Sciences, 100 Discovery Boulevard, Newark, DE 19713 USA; 5 Department of Veterinary Integrative Biosciences, Texas A&M University, College of Veterinary Medicine and Biomedical Sciences, 4458 TAMU, College Station, TX 77843 USA

**Keywords:** Environmental justice, Self-rated health, Mental health, Physical health

## Abstract

The Greater Fifth Ward (GFW) is a Northeast Houston, Texas, neighborhood with a legacy of industrial contamination and a confirmed cancer cluster. To understand self-rated health in the GFW, community-based participatory research (CBPR), was used to promote the inclusion of all partners. CBPR involves the community during each stage of the research process from design to research dissemination. A complete census was conducted, and 114 surveys were obtained in the environmental justice (EJ) community from July to November 2021. EJ communities shoulder an unfair burden of environmental exposures, pollution, and poor built environments. Mental and physical health were measured using the validated 12-item Short Form Health Survey (SF-12v2). We posited that the Black or African American (Black/AA) community would have lower mental composite scores (MCS) and physical composite scores (PCS) compared to the nation and their White counterparts. The MCS and PCS were calculated and compared against the national mean. Overall, participants had higher MCS and lower PCS than the national mean. Black/AA males and females had lower MCS compared to their White counterparts. White females had the lowest PCS among all respondents, significantly lower than the national average. MCS was lower among those who lived in the neighborhood longer. Burdens from pollution may impact residents’ health and perceived health. Targeted interventions or programs that improve mental or physical health would benefit this community and other inequitably burdened neighborhoods.

**Table:** 

Text box 1 Contributions to the literature
• There is limited research about mental and physical health scores in environmental justice communities.
• Public health policies addressing mental and physical health are needed in communities that experience cumulative environmental and non-environmental stressors.

## Introduction

Marginalized communities share a disproportionate burden of environmental pollution, natural hazards, and anthropogenic risks [1]. Many socioeconomically disadvantaged populations experience higher rates of morbidity and mortality that may be attributable to the cumulative effects of environmental stressors [2]. The relationship between poverty, race, life stages, and health is complex but a well-defined reality. Decades of research highlight the social inequalities of residential areas in proximity to highly industrialized areas, toxic exposures, and associated adverse health risks, such as increased risks of specific cancers, obesity, high levels of lead exposure, and a high prevalence of asthma [3, 4]. Neighborhoods or communities that experience the cumulative impacts of environmental and non-environmental stressors are known as environmental justice (EJ) communities. An example, of this inequity is the Greater Fifth Ward (GFW) neighborhood where residents’ health outcomes are worsened by historic and ongoing exclusionary practices, policies, and land-use decisions [5, 6].

Environmental risk factors that disproportionately impact economically disadvantaged communities contribute to a wide range of adverse health outcomes. A cross-sectional study of low- and middle-income children in New York reported cumulative environmental risk exposure such as crowding, housing problems, and noise was greater among low-income children which contributes to greater health risks [7]. Minority populations in the United States consistently report poor self-rated health more often than their White counterparts [8]. In another study conducted in a majority Hispanic fence-line community of Houston, a resident’s self-reported physical health was negatively correlated with the length of time a resident lived in the neighborhood [9].

Improving individuals’ health and environmental conditions across the socioeconomic continuum requires empirical evidence of health inequalities within these communities. The importance of characterizing and seeking sustainable solutions is underscored within EJ communities as hazard conditions are likely to increase in the coming decades due to the global impacts of climate change, meaning our current approaches are likely inadequate for this changing landscape [10]. At particular risk are communities within the nexus of legacy exposures and risks to natural hazard events, such as our target populations living within the Houston neighborhood of the GFW. While it is clear that the health of populations living in EJ communities is adversely impacted, less is known about the health impacts of living in these communities.

The GFW has experienced excess negative environmental exposures alongside an absence of positive environmental conditions such as parks [11]. These experiences are common within EJ communities the lack of adequate conditions inhibits a healthy life. While EJ research has made strides in highlighting environmental inequities and preventing the advent of new hazardous facilities in communities, it has faced challenges in addressing the mental and physical health of residents living within these communities. The study aims to improve our understanding of mental and physical health by comparing the GFW community to the national average and assessing the mental and physical health among different races and age groups. We hypothesized that the Black/AA residents of the GFW community would have lower mental composite scores (MCS) and physical composite scores (PCS) compared to the nation and their White counterparts. The validated 12-item Short Form Health Survey (SF-12v2) was used to evaluate mental and physical health. The SF-12v2 provides a reliable, validated, and efficient way to measure health outcomes in a largely underserved and understudied community.

## Materials and methods

### Study location

**Fig. 1:**
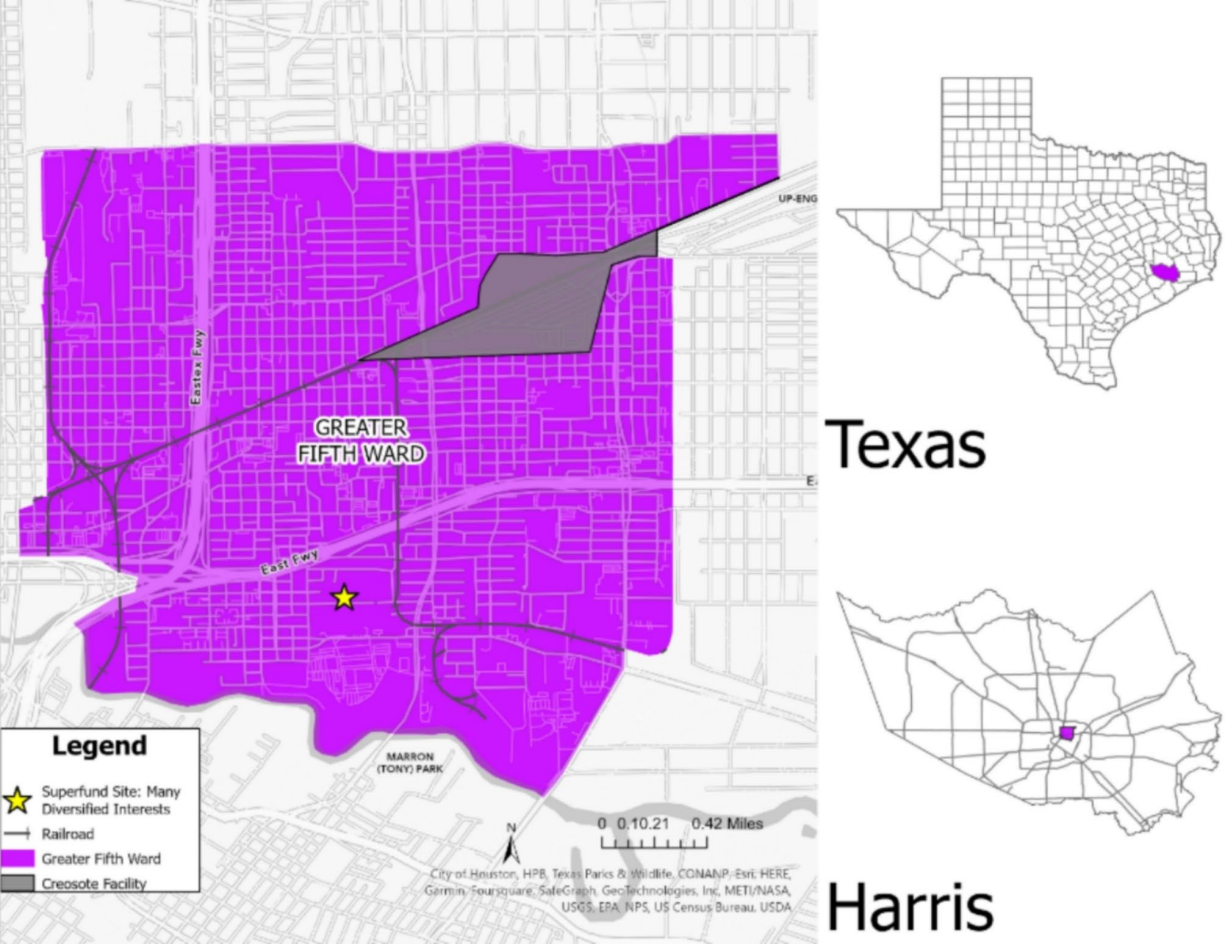
Study area of the Greater Fifth Ward Neighborhood, Houston, TX

Currently, the GFW neighborhood in Northeast Houston is primarily Hispanic and Black/AA (Fig. 1). It is one of the oldest communities in Houston and has a culturally rich history. Historically, the community has long been a majority Black/AA neighborhood, settled by freed slaves in the mid-1800s, and was thriving with railyard and industrial workers [12]. The GFW is within close proximity to a former creosote wood treatment facility, Houston Wood Preserving Works, which has potentially led to environmental contamination. The GFW is close to two Superfund sites on North and South Cavalcade Street that participated in known activities that lead to contaminated soil and groundwater with volatile organic compounds, polycyclic aromatic hydrocarbons, and various metals such as arsenic, chromium, copper, lead, and zinc [13, 14]. Another site, Many Diversified Interests, Inc. is a former foundry within the GFW boundary that operated from 1926 to 1992, the site’s groundwater is still being monitored by the U.S. Environmental Protection Agency (U.S. EPA) [15]. In 2020 and 2021, the Texas Department of State Health Services (DSHS) discovered and confirmed a cancer cluster in the GFW [16]. The DSHS investigations found higher rates of cancer (i.e., acute myeloid leukemia, lung, bronchus, esophagus, larynx, liver, and childhood acute lymphoblastic leukemia) than expected across 21 census tracks including the GFW [16, 17].

Today, the population of the GFW neighborhood is 96% minority and has a median income household income of $27,668 compared to the median household income of $52,338 in Houston [18]. Roughly, 12% of residents have obtained a bachelor’s degree. Even given these challenges, two community organizations, the Coalition for Community Organizations (COCO) and Impact have been deeply involved with community engagement, working with residents to improve public health and neighborhood conditions. The organizations helped establish the GFW block captains, who are neighborhood leaders who work to make their neighborhood livable and safe from environmental hazards, and contaminants. The GFW block captain program was modeled after the Houston Health Department’s Block Captain Network and was leveraged by COCO to meet the needs of their community. Thus, the longstanding partnership between the research team and the community organizations provided an opportunity to better understand and identify the community’s mental and physical health concerns related to existing vulnerabilities and helped establish relationships with local community-based EJ organizations.

### Survey instrument

The survey instrument contained four sections. In the first section, respondents were asked to provide demographic information (e.g., gender, race/ethnicity, and age), and information about the number of years they lived in their homes and neighborhood. The second section recorded information about the respondent’s health history and included questions about chronic illness (e.g., kidney or liver damage diagnosis, seizures, mental health concerns, mental confusion, etc.), cancer diagnoses, and self-rated physical and mental health. The SF-12v2 is a validated questionnaire to predict mental and physical health outcomes of populations and was shown to be reliable in the U.S., in low socio-economic communities, and different languages [19]. The third section pertained to behavioral and health habits such as smoking, alcohol, and drug use. The last section of the survey was used to gauge participants’ perceptions of environmental concerns and ascertain the respondent’s awareness of the chemical creosote and their knowledge of the joint Houston Health Department and DSHS investigation into cancer clusters in the neighborhood.

### Data collection

A complete census was conducted in the GFW neighborhood between July and November 2021. Community partnerships were developed with the COCO and Impact approximately one year before the project began. The GFW block captains were instrumental in the process and were involved with survey design, surveying, and research translation. The survey teams consisted of three individuals: a graduate assistant, a GFW block captain, and at least one individual who was fluent in Spanish. The GFW block captains, along with Texas A&M University students recruited from the Texas A&M University School of Public Health EpiAssist program [20, 21], received face-to-face training, online training, and pre-recorded training videos in addition to research ethics and compliance training. The Texas A&M University students and GFW block captains received training each day before the fieldwork began. The trained survey teams walked every public road and passed every home within the geographic boundary of the GFW during twelve sampling campaigns. Homes that were completely fenced off, abandoned, or deemed unsafe by the survey teams were not approached during canvassing. Participants received a $10 gift card for participating in the survey. The survey was approved by the Texas A&M University Institutional Review Board (IRB2021-0357D).

### Data analysis

Descriptive statistics were calculated for each variable, including demographics. Race was coded as either Black/AA or White. Asians, American Indian/Alaska Natives, Native Hawaiian or Pacific Islanders were excluded from the study to account for the low number of respondents in these groups. Hispanic/Latino/a were included in the demographics table to present a holistic view of the neighborhood’s ethnicity but were not included in the other analysis. Educational attainment was coded as less than a high school degree, high school diploma or equivalent, college degree or none. The respondents’ mean age and years lived in the neighborhood were calculated and reported in years. Their preferred language was coded as either English or Spanish. Responses to the SF-12v2 questions were used to estimate a mental composite score (MCS) and physical composite score (PCS) for each subject on a 0- to 100-point scale using methods developed by Ware et al. (2001) [22]. The SF-12v2 uses a norm-based algorithm and produces a composite score for self-reported mental and physical health between 0 and 100, and a mean value normalized to 50 which allows for comparison between groups in the study population and national averages [22]. MCS and PCS values for respondents were calculated and compared to the national mean using two-tailed t-tests. A complete case analysis was used, so that covariates with missing values were dropped before analysis. Multiple linear regressions were used to assess the impact of gender, age, race, and time living in the neighborhood on MCS and PCS. Statistics were calculated using STATA 17 SE (College Station, TX: StataCorp, LLC).

## Results

Between July and November 2021, twelve sampling campaigns yielded 114 completed surveys. Of those that responded 60.0% were female and 40.4% identified as male. Of the respondents, 18.4% were completed by White individuals, and 81.6% were completed by Black/AA individuals. Of those who responded, 87.7% were not Hispanic/Latino/a, and 12.3% were Hispanic/Latino/a. Of those who responded 16.7% of respondents had less than a high school degree, 47.4% had a high school diploma or equivalency, 32.5% had a college degree, and 3.5% had no education. English was the preferred language for 97.4% of survey respondents and Spanish was preferred by 2.6%. The mean age of respondents was 56 (SD = 18.2%). The mean years lived in the neighborhood was 32 years (SD = 25.6%) (Table 1).

**Table 1:** Demographic distribution of study participants in the Greater Fifth Ward neighborhood of Houston, TX, 2021

Characteristic	*n* (%)		
Sex			
Male		46 (40.3%)	
Female		68 (60.0%)	
Race			
Black/African American		93 (81.6%)	
White		21 (18.4%)	
Hispanic/Latino/a			
No		100 (87.7%)	
Yes		14 (12.3%)	
Education			
Less than High School		19 (16.7%)	
High School Diploma or Equivalency		54 (47.4%)	
College Degree		37(32.5%)	
No Education		4 (3.5%)	
Age in Years			
Mean (SD)		56 (18.2)	
Language			
English		111 (97.4%)	
Spanish		3 (2.6%)	
Years Lived in the Neighborhood			
Mean (SD)		32 (25.6)	

*N* = 114; SD = Standard Deviation

Respondents had a slightly higher MCS compared to the U.S. mean of 50 among all categories. However, Black/AA males had a lower mean MCS of 50.51 (95% CI: 47.50, 53.51; *p* = 0.72) compared to other groups, and Black/AA females had a MCS of 50.90 (95% CI = 48.45, 53.35; *p* = 0.47). Overall, males had a MCS of 51.73 (95% CI: 49.58, 53.87; *p* < 0.01), females had a MCS of 51.02 (95% CI: 49.15, 52.90; *p* = 0.14) and white females had a mean of 51.76 (95% CI: 46.64, 56.89; *p* = 0.47) categories, this seemingly contradictory finding has been witnessed within other tight-knit EJ communities in Houston, TX [9]. White males had a MCS of 54.08 (95% CI: 51.13, 57.04, *p* < 0.01) the highest MCS among the groups and compared to the national average MCS.

All categories investigated had PCS lower than the national mean for PCS. The PCS for males was 41.55 (95% CI: 40.85, 43.02; *p* < 0.001) and for females was 42.20 (95% CI: 41.24, 43.15; *p* < 0.001), significantly lower than the national mean scores. White males (41.33, 95% CI: 38.24, 44.42; *p* < 0.001) and White females (40.84, 95% CI: 38.29, 43.48; *p* < 0.001) both had significantly lower mean PCS compared to the national mean. Black/AA males (41.21, 95% CI: 39.32, 43.10; *p* < 0.001 and Black/AA females (42.58, 95% CI: 41.20, 43.96; *p* < 0.001) had a significantly lower mean PCS compared to the national mean (Table 2).

**Table 2:** Two-tailed t-test of mean values of mental and physical composite scores against national mean values in the Greater Fifth Ward neighborhood of Houston, TX, 2021

Variable	*n*	t value	Mean	95% Confidence Interval	*p*-value
**Mental composite score**					
Male	46	1.61	51.73	49.58–53.87	< 0.01*
Female	68	1.08	51.02	49.15–52.90	0.14
White male	9	3.19	54.08	51.13–57.04	< 0.01*
White female	12	0.76	51.76	46.64–56.89	0.23
Black/African American male	37	0.34	50.51	47.50–53.51	0.72
Black/African American female	12	0.74	50.90	48.45–53.35	0.47
**Physical composite score**					
Male	46	-11.50	41.55	40.08–43.02	< 0.001*
Female	68	-16.24	42.20	41.24–43.15	< 0.001*
White male	9	-6.46	41.33	38.24–44.42	< 0.001*
White female	12	-7.73	40.88	38.29–43.48	< 0.001*
Black/African American male	37	-9.43	41.21	39.32–43.10	< 0.001*
Black/African American female	12	-10.81	42.58	41.20–43.96	< 0.001*

*Statistically significant (p-value < 0.05)

When controlling for gender, age, race, and years lived in the neighborhood, those 66 to 79 years of age (coef. 8.45; 95% CI: 2.86, 14.03; *p* < 0.01) and those 80 and older (coef. 11.20; 95% CI: 3.96, 18.44; *p* < 0.01) were statistically significant and comparatively higher MCS compared to those 18 to 35. White study participants (coef. 2.93; 95% CI: -1.32, 7.18), had higher MCS compared to those who identified as Black/AA counterparts. Whereas living in the neighborhood longer was associated with a lower MCS (coef. -0.08; 95% CI: -0.16, 0.00) and was statistically significant *p* < 0.04. All other covariates in the MCS model were not statistically significant.

When controlling for gender, age, race, and years lived in the neighborhood, the older the participant the lower their PCS compared to younger participants. Those aged 36 to 65 had somewhat lower PCS compared to those 18 to 35 (coef. -2.41; 95% CI: -5.02, -0.08; *p* = 0.07), while those aged 66 to 79 had even lower PCS (coef. -3.50; 95% CI: -6.92, -0.08; *p* = 0.05). Finally, those 80 years old and older (coef. -5.44; 95% CI: -9.86, -1.01, *p* = 0.02) had comparatively lower PCS compared to all other ages. White participants had lower PCS compared to Black/AA participants, but it was non-statistically significant. All other covariates on PCS were not statistically significant (Table 3).

**Table 3:** Multiple linear regressions comparing the covariates age, gender, race, and years lived in the neighborhood on mental and physical composite scores (*N* = 114) of study participants in the Greater Fifth Ward neighborhood of Houston, TX, 2021

Variable	Coefficient	Standard Error	95% Confidence Interval	*p*-value
**Mental composite score**				
Sex				
Male (ref)	1.00			
Female	-1.21	1.61	-4.41–1.99	-0.75
Age				
18–35 years old (ref)	1.00			
36–65 years old	1.89	2.15	-2.37–6.14	0.38
66–79 years old	8.45	2.82	2.86–14.03	< 0.01*
80+	11.20	3.65	3.96–18.44	< 0.01*
Race				
Black/African American (ref)	1.00			
White	2.93	2.14	-1.32–7.18	0.18
Years Lived in the Neighborhood	-0.08	0.04	-0.16–- -0.00	0.04*
**Physical composite score**				
Sex				
Male (ref)	1.00			
Female	1.29	0.99	-0.66–3.25	0.19
Age				
18–35 years old (ref)	1.00			
36–65 years old	-2.41	1.31	-5.02–0.19	0.07
66–79 years old	-3.50	1.72	-6.92– -0.08	0.05*
80+	-5.44	2.24	-9.86– -1.01	0.02*
Race				
Black/African American (ref)	1.00			
White	-1.47	-1.31	-4.07–1.13	0.27
Years Lived in the Neighborhood	0.02	0.02	-0.05–0.07	0.43

*Statistically significant (p-value < 0.05)

## Discussion

While prior studies have reported that mental and physical health are commonly correlated [23, 24], survey respondents living in Houston’s GFW neighborhood reported higher than average MCS and lower than average PCS compared to the national average. The collective efficacy in the community may partially explain the higher-than-average MCS [25]. Collective efficacy occurs when a community builds social capital and community mobilization capacity among urban neighborhood residents in structurally disadvantaged communities [25]. People who are more civically involved in their communities report higher levels of collective efficacy [26]. Their involvement in community-centered activities is likely contributing to the neighborhood’s collective efficacy. The GFW block captains demonstrated this level of level involvement in their communities by organizing and hosting events, in addition, to their project-related work. The GFW block captains also received extensive training on environmental sampling, and surveying, and were well-equipped to answer community concerns about environmental contaminants of concern, skills training, and education have been shown to build social capital, civic engagement, and feelings of empowerment [27].

Despite higher mental conditions within older age brackets, there was a slight, but statistically significant reduction in MCS for those who lived in the neighborhood longer (Coef. -0.08, CI: -0.16, -0.00, *p* = 0.04). This suggests a potential environmental or social factor within the community that could negatively impact mental health.

Alternatively, the nearly two-standard deviation reduction in PCS scores in the community may be explained by several factors including low socioeconomic status, structural racism, behavioral actions, and chemical exposures. For many, socioeconomic status is the strongest predictor of health [28, 29], however, structural racism is also a major driver of poor health outcomes [30]. Structural racism is – the accumulation of the ways societies foster racial discrimination through inequitable systems. Additionally, the current practice of evaluating chemical-by-chemical and source-specific assessments of risks does not accurately characterize the varied cumulative physical and social stressors experienced by EJ communities [31]. Combined, these stressors place an undue, and not well understood, burden on neighborhoods like the GFW.

It is well-documented that self-rated health is lower among minorities and people of low socioeconomic status, and it is also a known predictor of mortality, chronic disease, and health behavior [31]. There is conflicting evidence on whether race has an impact on MCS, yet studies that investigated racial discrimination showed a marked difference in MCS. A UK longitudinal household study found that ethnic minorities who reported racial discrimination at 1-time point had an MCS of 1.93 (95% CI: -3.31, -0.56) points lower than those who did not [32]. Two or more incidents of racial discrimination reduced MCS by 8.26 (CI: -13.33, -3.18) compared to those that did not experience it [32]. Yet, other studies using SF-12v2 have shown MCS is higher in Black/AA populations compared to population norms [24]. The authors note that a more positive response may have occurred since the mode of administration was via telephone. This study avoids that since the questionnaire was interviewer-administered in person, however, this mode of administration may have similar implications.

Studies investigating the relationship between gender and PCS are not clear and vary among study populations. For example, a cross-sectional study conducted in South Los Angeles found that among AA adults, AA females reported lower PCS [33]. Similarly, in another study examining gender differences and health-related quality of life, after adjusting for sociodemographic characteristics and other clinical factors, females had lower SF-12 PCS scores compared to males [β = −1.78, standard error (SE) = 0.87, *p* < 0.05] [34]. Conversely, another study found that males reported less severe symptoms of shortness of breath and lower PCS than females [35].

The results of this study were communicated to the GFW block captains, and they provided valuable insight on how to communicate this information to the wider GFW community during town halls. The GFW block captains stated that they were not surprised by the results and agreed that the community may have slightly higher MCS because they work to support each other and worse PCS due to access to health care.

There are several important limitations to this study. First, the survey was interviewer-administered, which may have resulted in response bias if the study participants rated their overall mental or physical health higher when speaking with an interviewer. The survey was also administered during the COVID-19 pandemic, which may have influenced the respondents’ feelings of isolation and/or limited their ability to seek physical health care, resulting in them reporting lower self-rated physical or mental health. Selection bias is possible if residents of homes that could not safely be approached were different from those who lived in the homes that were approached by an interview team. The cross-sectional nature of this study means no temporal relationships between exposures and outcomes can be assessed. Improving our understanding of the relationship between mental and physical health in EJ communities requires longitudinal research. Nevertheless, this information provides a baseline measure to compare in future research.

## Conclusion

Measuring self-rated physical and mental health in EJ neighborhoods is one approach to understanding the potential health impacts of chronic exposure to a variety of potentially negative stressors. Improving our understanding of mental and physical health among EJ populations can help better inform policymakers and public health researchers about the needs of these communities and provide a rationale for targeted interventions.

## Data Availability

No datasets were generated or analysed during the current study.
